# 155 Practicing the active video fighting game strengthen antioxidant defense system: a pilot study

**DOI:** 10.1093/eurpub/ckae114.100

**Published:** 2024-09-26

**Authors:** Hayriye Çakır-Atabek, Cihan Aygün

**Affiliations:** Eskisehir Technical University, Faculty of Sport Sciences, Eskisehir, Turkey; Eskisehir Technical University, Faculty of Sport Sciences, Eskisehir, Turkey

## Abstract

**Background and purpose:**

When exercise –aerobic or anaerobic– is performed regularly, the antioxidant defense system, which prevents oxidative stress or reduces its damage, becomes stronger. Recently many studies showed that active video games (AVGs) could be used as useful intervention tool to increase physical fitness status among healthy population. From this point, this study aimed to investigate the acute and chronic effects of AVGs on indices of oxidative stress in young participants.

**Methods:**

Eleven healthy, physically active young males (age: 20.90±1.37 years, height: 177.41±5.68 cm, weight: 70.09±8.15 kg, and BMI: 22.22±1.91 kg/m2) volunteered to participate in the study. Major exclusion criteria were using tobacco products, and suffering from acute or chronic diseases. The participants performed a fighting AVGs -“Fighters Uncaged”- with Xbox Kinect console, in which the opponents were randomly selected, for 20 minutes per day, three days a week, for 4 weeks. The previous practices shown that this fighting AVGs provides vigorous physical activity (MET>7). At the beginning and at the end of the study maximum oxygen consumption (VO2max) was determined to verify the effectiveness of AVGs. Blood samples were obtained just before (after 10-min rest) and immediately after the VO2max tests. Oxidative stress was evaluated with total antioxidant status (TAS), total oxidant status (TOS), oxidative stress index (OSI), and total oxidized guanidine (TOG), which is an indirect marker of DNA damage, additionally, superoxide dismutase (SOD) was analyzed. Data were analyzed with dependent t-test and significance was accepted as p < 0.05.

**Results:**

The 4-week AVGs practicing significantly increased the VO2max (56.51±4.41 vs 58.52±3.66 ml/kg/min; p = 0.034). When investigate the acute effects, no significant alteration was determined between pre and post-test values for SOD, TAS, TOS, OSI, and DNA damage (p > 0.05). To determine the chronic effects, the pre-test values were compared, and result showed that SOD significantly increased (3.37±1.7 vs 7.65±4.6 U/ml; p = 0.022) and DNA damage significantly decreased (10.28±1.9 vs 7.75±1.7 ng/ml; p < 0.001) following 4-week AVGs practice.

**Conclusions:**

This study’s findings support the thought that AVGs can be an effective tool to enhance physical fitness status and strengthen the antioxidant defense system.

